# Acceptance of oral chemotherapy in breast cancer patients - a survey study

**DOI:** 10.1186/1471-2407-11-129

**Published:** 2011-04-12

**Authors:** Sarah Schott, Andreas Schneeweiss, Judith Reinhardt, Thomas Bruckner, Christoph Domschke, Christof Sohn, Michael H Eichbaum

**Affiliations:** 1 University Hospital Heidelberg, Department of Gynecology and Obstetrics, The National Center for Tumor Diseases, Voßstraße 9, D-69115 Heidelberg, Germany; 2 Institute for Medical Biometry and Informatics, Im Neuenheimer Feld 305, D-69120 Heidelberg, Germany

## Abstract

**Background:**

Oral (p.o.) chemotherapy treatments gained increasing importance in the palliative treatment of metastatic breast cancer (MBC). Aim of this survey was to evaluate the acceptance of p.o. treatment and patients' individual attitudes towards it.

**Methods:**

A specific 14 item-questionnaire was designed. Patients suffering from breast cancer receiving a newly launched p.o. or i.v. chemotherapy treatment were prospectively evaluated during 4 months of time. 224 questionnaires using descriptive statistics, chi-square test, Spearman correlation were evaluated.

**Results:**

Patients' median age was 54 years, 164 received i.v., 60 p.o therapy. 89% with p.o. and 67% with i.v. regimens would choose p.o. over i.v. therapy, if equal efficacy is guaranteed. Significant differences were especially found in terms of personal benefit (55% i.v., 92% p.o.), reduced feeling of being ill due to p.o. treatment (26% i.v., 65% p.o.), better coping with disease due to p.o. therapy (36% i.v., 68% p.o.). Side effects were significantly less often reported under p.o. treatment (19% p.o. vs. 53% i.v.)

**Conclusion:**

P.o. chemotherapy shows a high acceptance in MBC patients under palliative therapy. Compliance can be achieved in particular through a differentiated indication, patient education and competent support along a p.o. treatment.

## Background

Breast cancer is the leading neoplastic malignancy among women worldwide affecting one in 8 women [1]. Its incidence is steady and so is the number of patients under therapy with a chronic metastatic disease [2,3]. Therefore, the search for new innovative chemotherapeutic standards is ongoing. On the one hand, facing the demand to develop new drugs with higher antitumor activity and lower systemic toxicity [3]. On the other hand aiming to find drugs that control cancer as a chronic process and match requirements of a convenient long term application with high quality of life [4-6]. Conventional anticancer chemotherapy is dominated by complex intravenous (i.v.) regimes which affect patients' life considerably [7-9]. Not only the substantial amount of time spent for the treatment as such places a major burden on patients but also the frequent placement of indwelling catheters and its associated fears or severe complications [10-12]. Thus, the development of an oral (p.o.) anticancer therapy has been in focus over the past few years [13-17] and has also been evaluated in all-oral combination regimes [18]. Oral chemotherapy offers several benefits in terms of convenience, ease of administration, improved quality of live and economic aspects [9,19-23].

Previous surveys and studies have addressed the acceptance of oral chemotherapy among patients and health care professionals [24,25]. These results revealed that most patients, who had experienced p.o. therapy, favor p.o. over i.v. chemotherapy [8,19,24-30]. Since these data have been published an increasing number of oral chemotherapeutics have been licensed and approved and will find their way in adjuvant therapies. Our survey study investigates breast cancer patients' personal concerns, doubts and general feelings towards p.o. application as well as the patient's reasons for preferring a p.o. or an i.v. treatment in the adjuvant as well as palliative setting. The assessment of the study provides insights for health care professionals on patient orientated education as well as guidelines to obtain a high compliance under oral treatment. In addition, the study offers important information on patient desires as well as on prejudices that have to be taken into account when establishing oral chemotherapy as an alternative standard to i.v. treatment in palliative as well as adjuvant setting.

## Methods

### Patients

This study was conducted in accordance with the ethical principals laid down in the Declaration of Helsinki and the International Conference on Good Clinical Practice. The study protocol was approved by the Ethics Committee of the University of Heidelberg. All patients provided written informed consent before study entry.

Patients were eligible if they were ≥18 years old, had histologically confirmed breast cancer, currently either under i.v. or oral chemotherapy and if they provided written informed consent before in-clusion. Patients who had only received a prior hormonal therapy or those being not legally capable could not be included. Concomitant hormonal chemotherapy was not allowed.

### Questionnaire

The data published by Catania at al. [24] on the perception of oral chemotherapy and recent steps in p.o. drug development (covering MEDLINE results from 1969 to 2008) provided the basis for the preparation of our questionnaire [Additional file 1]. Their described validated questionnaire was transformed to our institutional standards and translated into German language (appendix 1). It evaluates the acceptance of p.o. versus. i.v. chemotherapy based on three different levels, focusing on personal, functional and tolerability-/efficacy-associated aspects.

### Statistical analysis

The absolute and relative frequencies with regard to the available response categories/response options were evaluated overall and differentiated by p.o. and i.v. treatment; this evaluation included the analysis of missing answers. For each of the questions 1-9 the frequency-distribution over the response-categories was analyzed graphically. The correlation of responses between questions 1 to 9 was assessed pairwise using the Spearman's rank coefficient. The association between two categorical variables was analyzed using the χ-squared test. Potential differences in patients' responses according to age group and route of administration were also examined using χ-squared test. Level of significance α was set to 5%.

## Results

### Patient characteristics

224 patients completed the questionnaire. 164 (73.2%) and 60 (26.8%) patients received i.v. and p.o. chemotherapy, respectively.

95 (57.9%) and 10 (6.1%) of the i.v. treated patients received the observed treatment in adju-vant and neoadjuvant setting, respectively. The remaining 59 (36.0%) patients were treated with palliative intent. Women undergoing palliative treatment were pretreated with a median of 3 prior systemic anticancer regimens in the oral group and 4.0 in the i.v. group. 20 (12.2%) patients of the i.v. group had experienced oral chemotherapy before, 12 (20.0%) of the p.o., respectively.

All 60 (100%) patients of the p.o. treated group received the observed treatment in palliative setting. These patients were pretreated with a median of 3.0 prior systemic anticancer regimens. 12.0 (20.0%) patients had already experienced oral chemotherapy before. 39 (65.0%), 17 (28.3%), 4 (6.7%) of the p.o. group were undergoing monochemotherapy, combination with i.v. or combination with another p.o. agents at the time of observation, respectively.

The mean age of the total studied population was 53 years (i.v. 52.6 years; p.o. 55.6 years), most patients graduated from middle school, were married and non-working. There were no relevant differences between the two treatment groups. Table 1 provides an overview on the main patient charac-teristics.

**Table 1:** Baseline characteristics (N = 224)

(N, % of group)	**i.v**.	**p.o**.
Number of participants	164	60

Mean age in years (range)	52.6 (30 - 76)	55.8 (26 - 81)

Treatment at the time of observation		

Neoadjuvant	10 (6.1%)	0

Adjuvant	95 (57.9%)	0

Palliative	59 (36.0%)	60 (100%)

Prior treatments		

Prior p.o. treatment	20 (12.2%)	12 (20.0%)

Number of prior systemic anticancer regimens when treated in palliative intent [mean (range)]	4.0 (1 - 9)	3.0 (1 - 10)

Level of education		

Lower school	43 (26.2%)	15 (25.0%)

Middle school	69 (42.1%)	25 (41.7%)

High school	21 (12.8%)	7 (11.7%)

University graduation	29 (17.7%)	13 (21.7%)

Missing information	2 (1.2%)	0

Active employment	62 (37.8%)	20 (33.3%)

Marital status		

Unmarried	33 (20.1%)	12 (20.0%)

Married	123 (75.0%)	42 (70.0%)

Widowed	8 (4.9%)	6 (10.0%)

### Questionnaire

**Figure 1:**
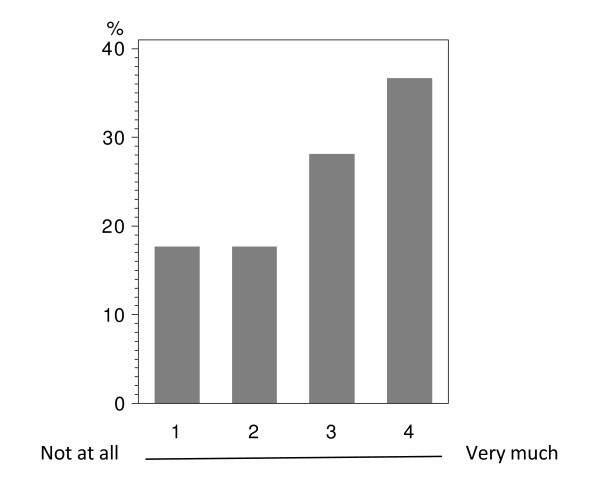
**Question 1: Do you see a personal benefit receiving oral instead of i.v. treatment? (evaluable patients; N = 221)**.

**Figure 2:**
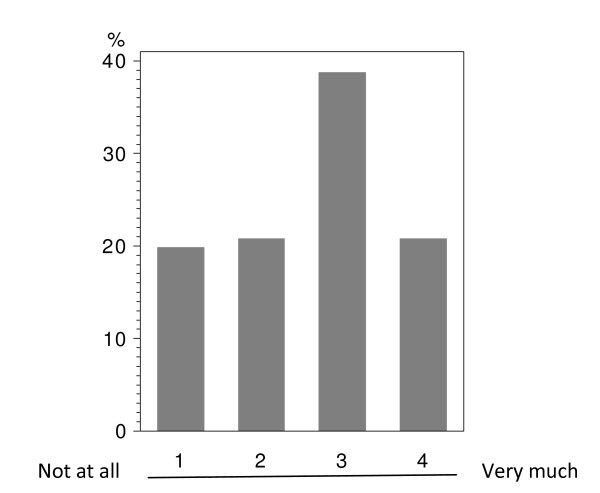
**Question 7: To which extend has your everyday life been affected in the past by hospital visits performed especially due to i.v. administration of chemotherapy? (evaluable patients; N = 217)**.

**Figure 3:**
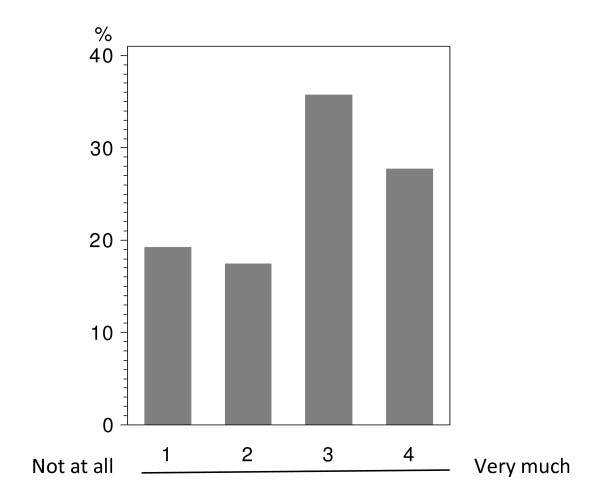
**Question 4: Do you believe that an oral chemotherapy affects your everyday life and your family surrounding less than an i.v. chemotherapy? (evaluable patients; N = 224)**.

**Figure 4:**
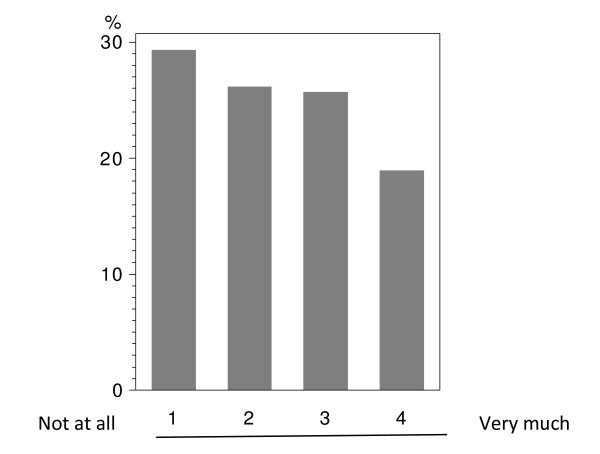
**Question 8: Do you believe than an oral chemotherapy could make it easier for you to cope with your disease? (evaluable patients; N = 222)**.

**Figure 5:**
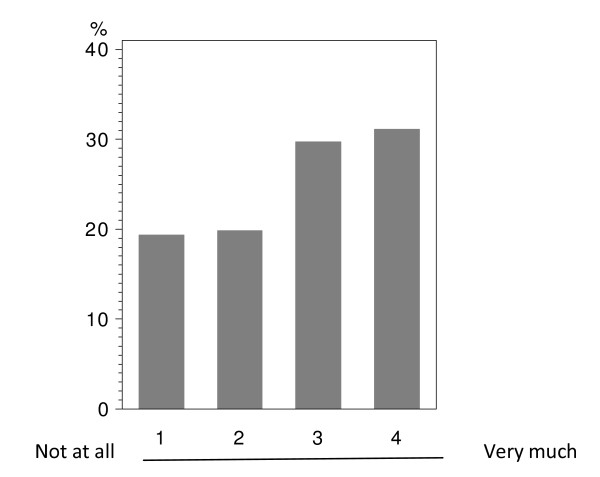
**Question 9: Do you think that a chemotherapy with capsules or tablets would make it easier for you to handle your disease by giving you more autonomy outside the clinic? (evaluable patients; N = 222)**.

**Figure 6:**
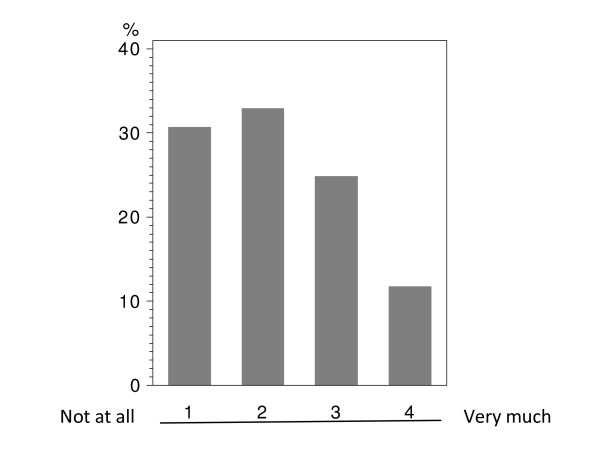
**Question 2: Do you believe that you feel less ill when receiving oral instead of i.v. chemotherapy? (evaluable patients; N = 222)**.

**Figure 7:**
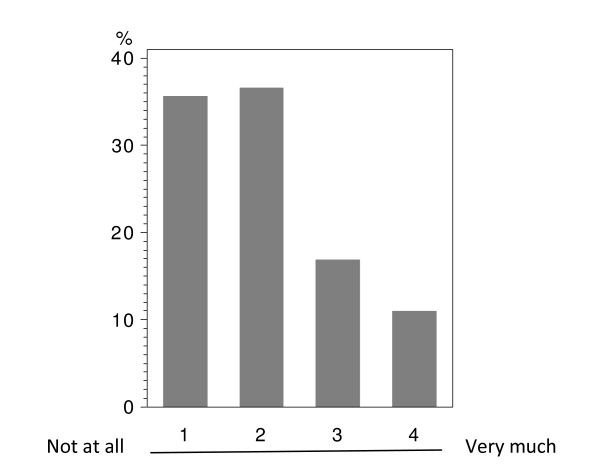
**Question 6: Do you believe that an oral chemotherapy has less side effects than an i.v. chemotherapy? (evaluable patients; N = 219)**.

**Figure 8:**
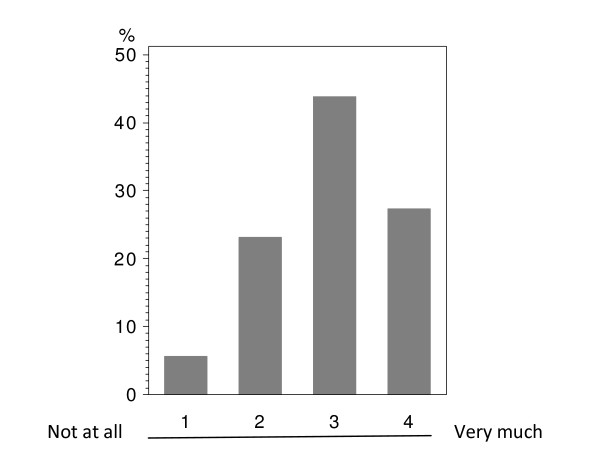
**Question 5: Do you believe that an oral chemotherapy is as effective as an i.v. chemotherapy? (evaluable patients; N = 212)**.

**Figure 9:**
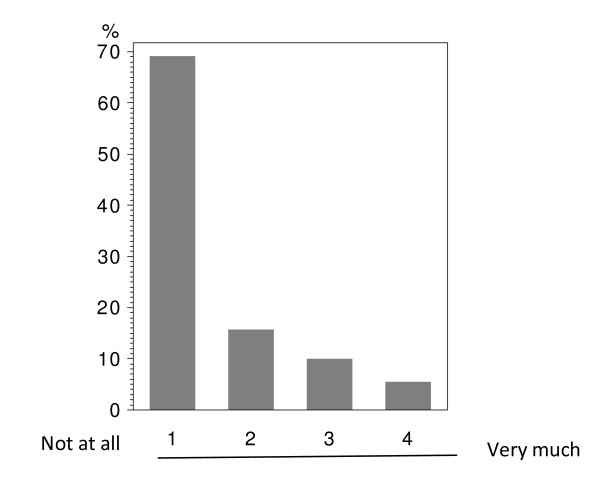
**Question 3: Are you afraid that - in case of an oral chemotherapy - you could eventually take the capsules/tablets in a wrong way? (evaluable patients; N = 223)**.

In total 224 patients answered the questionnaire correctly (i.v.: 164; p.o.: 60), with analyzable answers per question ranging from 212 to 224 (i.v.: 155-164; p.o.: 56-60; see Table 2 and Figure 1, 2, 3, 4, 5, 6, 7, 8, 9). Question 1 to 9 showed a broad range of answers as displayed in the bar graphs provided in Figures 1, 2, 3, 4, 5, 6, 7, 8, 9. Partially significant differences were observed between the p.o. and i.v. group (Table 2). 48 patients documented free-text com-ments (question 14) providing additional explanations. Regarding questions 12 and 13 over 50% of cases were answered in an invalid way, so data from these questions are not analyzed.

**Table 2:** Results of Questions 1 - 9

**Question No**.	Therapyform	N evaluable patients	1 not at all	2	3	4 very much	p-value
1	i.v.	161	22.98	22.36	26.09	28.57	<0.0001
	
	p.o.	60	3.33	5.00	33.33	58.33	

2	i.v.	162	35.80	38.27	20.37	5.56	<0.0001
	
	p.o.	60	16.67	18.33	36.67	28.33	

3	i.v.	163	65.03	17.18	10.43	7.36	0.0757
	
	p.o.	60	80.00	11.67	8.33	0.00	

4	i.v.	164	23.17	20.12	32.32	24.39	0.0074
	
	p.o.	60	8.33	10.00	45.00	36.67	

5	i.v.	155	7.10	29.68	43.87	19.35	<0.0001
	
	p.o.	57	1.75	5.26	43.86	49.12	

6	i.v.	160	40.63	40.63	13.13	5.63	<0.0001
	
	p.o.	59	22.03	25.42	27.12	25.42	

7	i.v.	161	21.12	20.50	39.75	18.63	0.5605
	
	p.o.	56	16.07	21.43	35.71	26.79	

8	i.v.	163	36.81	26.99	22.09	14.11	<0.0001
	
	p.o.	59	8.47	23.73	35.59	32.20	

9	i.v.	164	23.78	23.17	28.05	25.00	0.0005
	
	p.o.	58	6.90	10.34	34.48	48.28	

[% of patients in the i.v. (N = 164) and p.o. (N = 60) population]

#### Personal aspects

Patients' personal benefits were addressed with questions 1, 4 and 7.

In the overall population, including oral and i.v. treated patients; there was a tendency in favor of oral treatment with regard to the question, if the patient sees a personal benefit when receiving oral instead of i.v. treatment (Figure 1). This direct personal benefit due to oral chemotherapy was judged with a significant difference between both groups (p < 0.0001). Over half (58.3%) of the patients receiving p.o. treatment expected a clear benefit from the oral application form; while the corresponding percentage was 28.6% in the i.v. population (Table 2 - question 1).

The impact on everyday life due to clinical visits and hospitalizations caused by i.v. treatment (question 7) was assessed in both study groups with the majority of patients describing a medium to strong impact (categories 3+4: i.v.: 58.4%; p.o.: 62.5%); there was no significant difference between the administration routes (Table 2 - question 7). Moreover, there were no age-depending differences below or above 50 years within this group, if age was set as point of interest (data not shown). The overall population tended to see a moderate impact on the everyday life due to i.v. associated hospital visits (Figure 2).

The question whether daily and family life is impacted less by p.o. than by i.v. chemotherapy (question 4) revealed a significant difference (p < 0.0074) between the two treatment groups. In both groups a relevant percentage of patients believed in less disturbances due to p.o. treatment (p.o.: 36.7%; i.v.: 24.4%; Table 2 - question 4). While in the i.v. population 23.2% did not expect a relevant benefit from p.o. treatment with regard to their daily life, such response was obtained in only 8.3% in the p.o. population.

#### Functional aspects

The question how functional aspects in terms of coping with the disease are influenced by the application form was assessed (questions 8 and 9).

Question 8 addressed how therapeutic application could support the coping with disease in ge-neral. The overall population did not show a clear trend (Figure 4). 32.2% of the p.o. group saw a clear benefit due to oral chemotherapy; whereas only 8.5% expected no support at all through oral medication. A significant difference was observed when comparing the p.o. and i.v. groups (p < 0.0001). 36.8% of the i.v. treated patients did not see any positive aspect by p.o. chemotherapy with regard to better coping with breast cancer; only 14.1% strongly believed in a better coping when using orally administered drugs (Table 2 - question 8).

Question 9, asking if oral chemotherapy would make it easier for the patient to handle her disease by providing more autonomy outside the clinic, also showed a significant difference between both therapy groups (p = 0.0005). 48.3% of the patients with p.o. treatment believed in a better handling of their disease due to p.o. application. On the opposite, only 25.0% of the i.v. treated patients claimed a clear benefit by p.o. chemotherapy (Table 2 - question 9). The overall population slightly tended to expect more autonomy due to oral treatment (Figure 5).

#### Tolerability- and efficacy-associated aspects

Tolerability- and efficacy-associated aspects were addressed with questions 2, 3, 5 and 6.

The overall population showed a tendency not to believe in feeling less ill under oral compared to i.v. treatment (Figure 6). There was a significant difference in patients' subjective per-ception with regard to feeling less ill when treated orally or intravenously (p < 0.0001). While the i.v. patients tended to respond that oral treatment will not help them in coping with their disease, oral patients claimed a benefit from oral treatment (categories 3+4: i.v.: 25.9%; p.o.: 65.0%; Table 2 - question 2).

The perceptions concerning lower side effects under p.o. chemotherapy were again signifi-cantly diverse between both groups (p < 0.0001). Patients' opinions varied broadly within the p.o. treated group as such; 22.0% believed that there is no reduction of side effects due to p.o. chemotherapy while 25.4% expect such reduction. The i.v. treated patients were more skeptical: 40.6% did not expect reduction of side effects under oral treatment with only 5.6% being opposed to this opinion (Table 2 - question 6). In the overall population the majority of patients tended not to believe in fewer side effects due to oral administration (Figure 7).

A significant variation was assessed between both groups in terms of creed towards equal ef-ficacy of p.o. and i.v. drugs (p < 0.0001). Patients under oral treatment strongly believed in equal efficacy (49.1%). A major part of the patients receiving i.v. chemotherapy also assumed that there is an equivalent efficacy (category 3: 43.9%, category 4: 19.4%), however, also 29.7% (rank 2) of these patients were more opposed to this opinion (Table 2 - question 5). In the overall population the majority of patients tended to assume that there is an equivalent efficacy (Figure 8).

Correct intake of p.o. chemotherapy is a major issue for successful treatment. 80.0% of the p.o. and 65.0% of the i.v. treated patients were not concerned about a wrong intake (Table 2 - question 3). Therefore, the trend in the overall population was clear towards no hesitation about oral intake (Figure 9). Interestingly, there was no difference between the age groups if an age of 50 was set as a cutoff (data not shown); thus, elderly patients are not more reserved towards p.o. chemotherapy than the young.

#### Patients' preference and source of information

If patients could choose between an equally effective p.o. or i.v. application, 89.3% of the orally treated women would again choose p.o. treatment. Also 67.1% of those patients receiving i.v. treatment would prefer p.o. medication.

The patients specified the following sources with regard to information about the disease and treatment options: physicians (97.7%), internet (38.8%), second opinions (23.6%) and relatives (16.9%); there were no significant differences between both groups.

#### Correlations

The correlations between answers given to any set of two of the questions 1 to 9 was explored using the Spearman rank coefficient (Table 3). This analysis was not adjusted for multiple comparisons. Most of the correlations revealed significant p-values and - taken the low magnitude of the observed p-values - it is very likely that these would still reach the level of significance, even if adjustment for multiple comparisons would have been performed. Moderate correlations (r ≥ 0.4) were found for questions 1, 2, 4, 5, 6, 8 and 9. The largest coefficients (bold in Table 3) were found between questions 8 and 9 (r = 0.64), 1 and 8 (r = 0.62) as well as questions 4 and 9 (r = 0.60). Specifically, patients who strongly believed that oral chemotherapy could make it easier for them to cope with their disease (question 8) tended to strongly agree with the point that such chemotherapy would make it easier for the patients to handle their disease by providing more autonomy outside the clinic (question 9).

**Table 3:** Correlation between item 1 through 9 in the questionnaire

	Q1	Q2	Q3	Q4	Q5	Q6	Q7	Q8	Q9
Q1	1.00000	**0.57534** **<.0001**	0.26799 <.0001	0.53925 <.0001	0.43413 <.0001	0.36976 <.0001	0.34612 <.0001	**0.61854** **<.0001**	**0.55128** **<.0001**
	
Q2		1.00000	0.27215 <.0001	0.44921 <.0001	0.22798 <.0001	0.45738 <.0001	0.31618 <.0001	**0.54728** **<.0001**	0.48659 <.0001
		
Q3			1.00000	0.05066 0.2857	0.19717 <.0001	0.18867 <.0001	0.08223 0.0878	0.18750 <.0001	0.28425 <.0001
			
Q4				1.00000	0.18743 0.0001	0.21409 <.0001	0.37361 <.0001	0.44632 <.0001	**0.59938** **<.0001**
				
Q5					1.00000	0.23208 <.0001	0.18818 0.0001	0.35262 <.0001	0.34081 <.0001
					
Q6						1.00000	0.10478 0.0310	0.34833 <.0001	0.26770 <.0001
						
Q7							1.00000	0.36320 <.0001	0.39544 <.0001
							
Q8								1.00000	**0.63656** **<.0001**
								
Q9									1.00000

Spearman Correlation Coefficients

Prob > |r| under H0: Rho = 0

Q = question

## Discussion

Breast cancer - with its high incidence worldwide - requires effective treatment options with high practicability, selectivity and tolerability. At present most therapy options are complex i.v. treatments, being usually connected with traveling efforts, time-consuming hospital-procedures and high costs [23,31-33]. In contrast, p.o. chemotherapy can easily be applied in an out-patient setting, e.g. in cooperation with the patient's general practitioner, thus reducing hospital visits and burdensome invasive medical procedures, such as placements of catheters. It was already demonstrated that, with oral treatment, the time spend in hospital as well as the overall costs of chemotherapy can be reduced considerably [13].

However, patients' and health care professionals' skepticism towards p.o. treatment has been observed [9,11,19,24,26,34-36]. Hesitations towards p.o. drug prescriptions mainly resulting from apprehensions regarding bioavailability, doubts about efficacy, and concerns about compliance initially hampered the development of p.o. anticancer drugs [26,37]. In addition, patients sometimes believed that p.o. drugs are prescribed as last resort [24]. In the meanwhile, results from various studies contributed to the resolution of such prejudices and led to considerable progress in the development of oral treatment options [13,14,16,17] as well as of all-oral chemotherapy combinations [9,26,38-40].

When talking about oral chemotherapy, the therapy course and the therapeutically success first of all depend on patient-independent factors such as bioavailability, side effect profile (e.g. gastrointestinal toxicity) and optimal dosing schedules. Oral treatments face additional pathways such as gastrointestinal passage and first-pass effects in comparison to i.v. treatments and thus require specific knowledge of healthcare professionals [26]. The development of modern oral chemotherapies led to oral treatment options with adequate pharmacokinetic properties, acceptable side effect profiles and high efficacy [16,38,40-44].

Since 2005, when Catania et al. assessed patients' perceptions on efficacy of p.o. therapy, several oral agents have been licensed for breast cancer treatment and gained acceptance in this indication [30]. Based on the above mentioned efforts in oral drug development, initial reservation is increasingly obsolete and usage of p.o. treatment has grown. Among our interviewed patients, 9 out of 10 being treated orally and 7 out of 10 being treated intravenously would prefer p.o. over i.v. treatment if same efficacy is ensured. This is in agreement with the data of Liu et al, who also reported a high patient preference (89%) for p.o. treatment [19]. Other studies involving patients having experienced p.o. and i.v. treatment within the respective trial revealed patient preferences of 39% [45], 64% [20], 74% [25], 84% [46] and 90% [47]. Moreover, our survey revealed that the majority of patients in the p.o. and the i.v. group believed in equal efficacy of both routes of administration. However, p.o. treated patients trusted more in oral therapy than those under i.v. treatment, leading to a significant difference between both groups; in the i.v. group approximately 1/3 of patients still were of the opinion that i.v. drugs generally provide higher efficacy. Presumably, unfamiliarity with p.o. treatment led to these hesitations.

Commercial availability of active p.o. drugs will not directly ensure their use [26]. It is known that patients' compliance may still to some extent be hampered by general prejudices and incertitude con-cerning correct usage of the medication. Thus, oral chemotherapy will only be effective if compliance is optimized. For that reason, the ability of patients to understand the treatment in general, the specifics of an oral chemotherapy and the treatment schedule are key issues for a successful p.o. therapy. Our survey showed that the treating physicians still represent the main source for such treatment information, emphasizing their role for a correct therapy run. Data have shown that patients with p.o. chemotherapy treatments are generally motivated to ensure intake of their treatment [48,49]. Comparing recent studies (2000 - 2009) to those from the early 90's clear improvements in patient compliance have been observed [49]. An understandable treatment regimen and the use of patient dairies can reduce misunderstandings or wrong intakes [50,51]. Detailed information on side effect profiles (e.g. using patient information leaflets) and on requirements for treatment adaptations enable patients to contribute to the treatment and give them a greater sense of personal responsibility [26].

The impact of i.v.-treatment-related hospital visits on everyday life has been mentioned to be a major factor influencing quality of life [24]. In both treatment groups investigated in our survey the majority of patients agreed that oral treatment contributes to a better quality of life; especially those women who had experienced p.o. treatment confirmed a clear benefit (i.v.: 43%; p.o.: 63%). We assumed that especially younger women with young children would prefer p.o. therapy, however - consistent with the results of Catania et al. - no significant age-depended differences could be observed [24].

It is interesting to see that most of the questions analyzed with our survey revealed significant differences between the p.o. and i.v. group, indicating that prior treatment experience guides the acceptance of oral vs. intravenous therapy. This is also true with regard to the coping and handling of the disease. Approximately 50% of the women with p.o. treatment saw a clear benefit due to personal freedom and the possibility of self-management by p.o. application. Some women stated that a p.o. treatment gives them the feeling of a chronic instead of a life-threatening disease and allows them a better coping with the disease. The i.v. treated population was more skeptic. Some of the patients who had not yet experienced p.o. chemotherapy stated that they would miss the personal contact to other patients, the peer group benefit as well as the frequent interaction with the medical team. Most patients appreciated however, the possibility to reduce the daily presence of their disease by using oral treatment combined with a close connection to the centre and the availability of supporting staff via phone or out-patient clinic if required. The importance of this infrastructural setting was emphasized before [24]. It is important to mention that especially patients under adjuvant or neoadjuvant treatment stated that confrontation with other patients in a more progressed status of disease causes high emotional involvement; therefore such patients often prefer a home-based treatment. Also to mention the institutional triggered side effects such as psychogenic nausea along with institution-induced vomiting that considerably reduces quality of life and often seems to get worse with longer duration of palliative i.v. chemotherapy. Those negative issues are not relevant when using p.o. chemotherapy.

In general, the major benefit of oral versus i.v. chemotherapy can be described with a broader flexibility enabling a more individualized therapy and a higher quality of life. However, the amount of tablets or capsules needed to be swallowed has to be taken into account. Compliance can be lower if patients have to swallow numerous tablets every day [34,35] and studies have shown that a daily intake of maximum 6-8 tablets is acceptable [9]. This finding should be taken into account in the future development of oral drugs. One example of such development are so called duplex drugs, establishing a chemical link between two highly active anticancer compounds and resulting in a new, mono-molecule'[52].

## Conclusion

Oral chemotherapy plays an increasing role in breast cancer therapy. So far, it has mainly be-en used in palliative setting, however it will gain its place additionally in adjuvant settings as the PARPi inhibitors will show. High efficacy, adequate tolerability, acceptable bioavailability with low inter- and intra-individual variability and a limited number of tablets/capsules per day are basic prerequisites and have been limiting factors for oral drug development. When fulfilling these requirements, oral treatment can contribute to an improvement of the patients' quality of life, which remains high priority especially in metastatic disease. Nevertheless, successful oral therapy requires compliance. Thus, a differentiated indication as well as a detailed patient-oriented education and explanation of the p.o. treatment schedule, including information on potential side effects, are key factors for a successful treatment.

## Competing interests

The authors declare that they have no competing interests.

## Authors' contributions

SS, AS, CS, ME, TB were involved in the conception, discussion and design of the study. SS, CD, JR, ME involved in the provision of study material, patients, performed the survey, gained data. SS, JR, CD, ME, TB performed the data analysis and interpretation. SS and ME wrote the manuscript and coordinated the study. All authors read and approved the final version.

## Pre-publication history

The pre-publication history for this paper can be accessed here:

http://www.biomedcentral.com/1471-2407/11/129/prepub

## Supplementary Material

Additional file 1 **Questionnaire**. The questionnaire used for this survey is shown. Click here for file
