# Supplementary material for: Acceptance of oral chemotherapy in breast cancer patients - a survey study
Source: BMC Cancer. 2011 Apr 12;11:129. doi: 10.1186/1471-2407-11-129 (PMC3080835; doi:10.1186/1471-2407-11-129)
Supplement: Additional file 1 — Questionnaire. The questionnaire used for this survey is shown. [file 1471-2407-11-129-S1.DOC]

**Questionnaire**

1. Request of general information concerning age, education, job, family situation
2. Current Chemotherapy – p.o. or i.v.
3. Questions concerning the option to receive oral chemotherapy.

(Questions 1-9; option to chose from 1 to 4, with “1 = not at all” and “4 = very much”)

1. Do you see a personal benefit receiving oral instead of i.v. treatment?
2. Do you believe that you feel less ill when receiving oral instead of i.v. chemotherapy?
3. Are you afraid that – in case of an oral chemotherapy – you could eventually take the capsules/tablets in a wrong way?
4. Do you believe that an oral chemotherapy affects your everyday life and your family surrounding less than an i.v. chemotherapy?
5. Do you believe that an oral chemotherapy is as effective as an i.v. chemotherapy?
6. Do you believe that an oral chemotherapy has less side effects than an i.v. chemo­therapy?
7. To which extend has your everyday life been affected in the past by hospital visits performed especially due to i.v. administration of chemotherapy?
8. Do you believe than an oral chemotherapy could make it easier for you to cope with your disease?
9. Do you think that a chemotherapy with capsules or tablets would make it easier for you to handle your disease by giving you more autonomy outside the clinic?
10. If you could choose between an oral and an i.v. chemotherapy, would you have chosen an oral therapy if both treatments show the same efficacy?

Yes or No

1. How do you inform yourself about your disease and treatment options?
   - 1. Treating physician
     2. Second opinion
     3. Internet
     4. Relatives
     5. Support group / patient organization,
     6. Others
2. Provide a ranking (from 1-5) of advantages of an oral chemo­the­rapy you would hope for at the most (“1 = most important benefit”; “5 = less important”)

- Better tolerance
- Easier application
- Less hospitalizations
- More personal freedom/independence
- Avoidance of vein punctures

1. Provide a ranking (from 1-5) of disadvantages of an oral chemotherapy you would fear at the most (“1 = most important fear”; “5 = less important”)

- Lower efficacy
- Wrong intake - intake too low
- Wrong intake - intake too high
- Interactions with other oral medications
- Fear of not being under medical control often enough

1. Do you have further suggestions and opinions that you like to provide concerning oral chemotherapy?
